# Factors Influencing the Recurrence Potential of Benign Endometrial Polyps after Hysteroscopic Polypectomy

**DOI:** 10.1371/journal.pone.0144857

**Published:** 2015-12-11

**Authors:** Jehn-Hsiahn Yang, Chin-Der Chen, Shee-Uan Chen, Yu-Shih Yang, Mei-Jou Chen

**Affiliations:** Department of Obstetrics and Gynecology, National Taiwan University Hospital and National Taiwan University College of Medicine, Taipei, Taiwan; University Medical Center of Princeton/Rutgers Robert Wood Johnson Medical School, UNITED STATES

## Abstract

**Background:**

An endometrial polyp is a frequently encountered gynecologic disease with abnormal uterine bleeding and infertility being the two common presenting problems, and hysteroscopic polypectomy is an effective method to remove them. The postoperative polyp recurrence might result in reappearance of abnormal uterine bleeding or infertility, whereas factors influencing the postoperative recurrence potential have limited data.

**Methods:**

This case-series report included 168 premenopausal women who suffered from endometrial polyps and underwent hysteroscopic polypectomy. All of them were awaiting a future pregnancy. Office hysteroscopy was done before and after hysteroscopic polypectomy, in which preoperative hysteroscopy examined the number, type, and location of endometrial polyps, and postoperative hysteroscopy checked the polyp recurrence. Surgical indications, either infertility or the presentation of abnormal uterine bleeding, and follow-up duration were recorded.

**Results:**

Seventy-three out of 168 (43%) women had polyp recurrence after hysteroscopic polypectomy. Multivariate logistic regression analysis revealed that more endometrial polyps (*P* = 0.015) and longer duration of follow-up (*P* = 0.004) were significantly associated with an increased risk of postoperative polyp recurrence. The type of endometrial polyps was not correlated with polyp recurrence potential, whereas pedunculated type endometrial polyps were closely related to the presentation of abnormal uterine bleeding (*P* = 0.001).

**Conclusions:**

A higher number of endometrial polyps and longer follow-up duration are associated with a greater potential of polyp recurrence after hysteroscopic polypectomy.

## Introduction

Endometrial polyps are benign localized overgrowths of endometrial tissue, composed of glands, stroma, and blood vessels covered by epithelium [1]. It is a frequently encountered gynecologic disease with abnormal uterine bleeding (AUB) being the most common presenting symptom. Endometrial polyps might also result in infertility by intracavitary bleeding or presenting an abnormal environment for embryo implantation [2].

Endometrial polyps can be diagnosed by ultrasound, sonohysterography, hysteroscopy and curettage. Among them, hysteroscopy is superior to the other three methods because it is able to detect the number, type, and location of endometrial polyps. After diagnosis, hysteroscopic polypectomy is now the gold standard for treatment. It is undertaken under direct visualization to completely remove the polyps with adjacent endometrium left intact [3]. For infertile women with no other reason to explain their infertility, hysteroscopic polypectomy appears to improve fertility and increase pregnancy rates [2,4,5,6,7].

Studies demonstrated the postoperative recurrence rates of endometrial polyps to range from 2.5% to 43.6%, depending on the follow-up duration and the nature of polyps [3,8,9]. Hyperplastic polyp without atypia has a higher risk of postoperative recurrence than that of benign polyps (43.6% vs. 8.3%), although benign polyps have a certain ability to recur [3]. The recurrent endometrial polyps might therefore result in reappearance of AUB or infertility [8].

Infertile women are more likely to suffer from endometrial polyps [10], which suggests a causative relationship between endometrial polyps and infertility [4]. However, it is difficult to explain why some women have a tendency to experience polyp recurrence and others do not. The recurrence of endometrial polyps might be due to the polypoid background in the endometrium resulting from genetic aberrations [11]. The factors influencing postoperative recurrence potential of benign endometrial polyps have limited data, and we aim to carry out the analysis in this study.

## Materials and Methods

### Subjects

We enrolled premenopausal women who had endometrial polyps and underwent hysteroscopic polypectomy. There was endogenous estrogen produced by these women. The diagnosis was endometrial polyp without hyperplasia exclusively made by histopathologic examination, in which there were irregularly dilated endometrial glands and thick-walled vessels scattered in fibrotic stroma. These women were awaiting a future pregnancy, though some of them did not plan a pregnancy at that time. Only those who underwent office hysteroscopy both pre- and postoperatively were included. Finally there were 168 women in this retrospective case-series report.

Their age, gravida, parity, surgical indications, and follow-up duration were recorded. Infertility was defined as inability of a couple having frequent intercourse and not using contraception to conceive within one year. Abnormal uterine bleeding was defined as prolonged menstrual bleeding more than seven days or the presentation of any intermenstrual bleeding. This study was approved by the Institutional Review Board (IRB) of the National Taiwan University Hospital. Since this descriptive study was done by reviewing medical records, obtaining the informed consent from participates was not mandatory according to the rules of IRB. The patient records/information was anonymized and de-identified prior to analysis.

### Office hysteroscopy

Office hysteroscopy was done in the follicular phase before hysteroscopic surgery for diagnosis and localization of endometrial polyps. It was carried out with Hysterovideoscope HYF type V (Olympus Optical Co., Tokyo, Japan). This flexible, single-flow hysterovideoscope has an outer diameter of 3.8 mm and a 120° field of view. The procedure has been described previously [12,13,14]. In brief, after application of the speculum and disinfection of the cervix, the hysterovideoscope was introduced intracervically. Neither anesthesia nor a tenaculum was needed. The distending media of 10% dextrose solution were delivered into the uterine cavity by simple gravity flow from 70 cm above the women.

**Fig 1:**
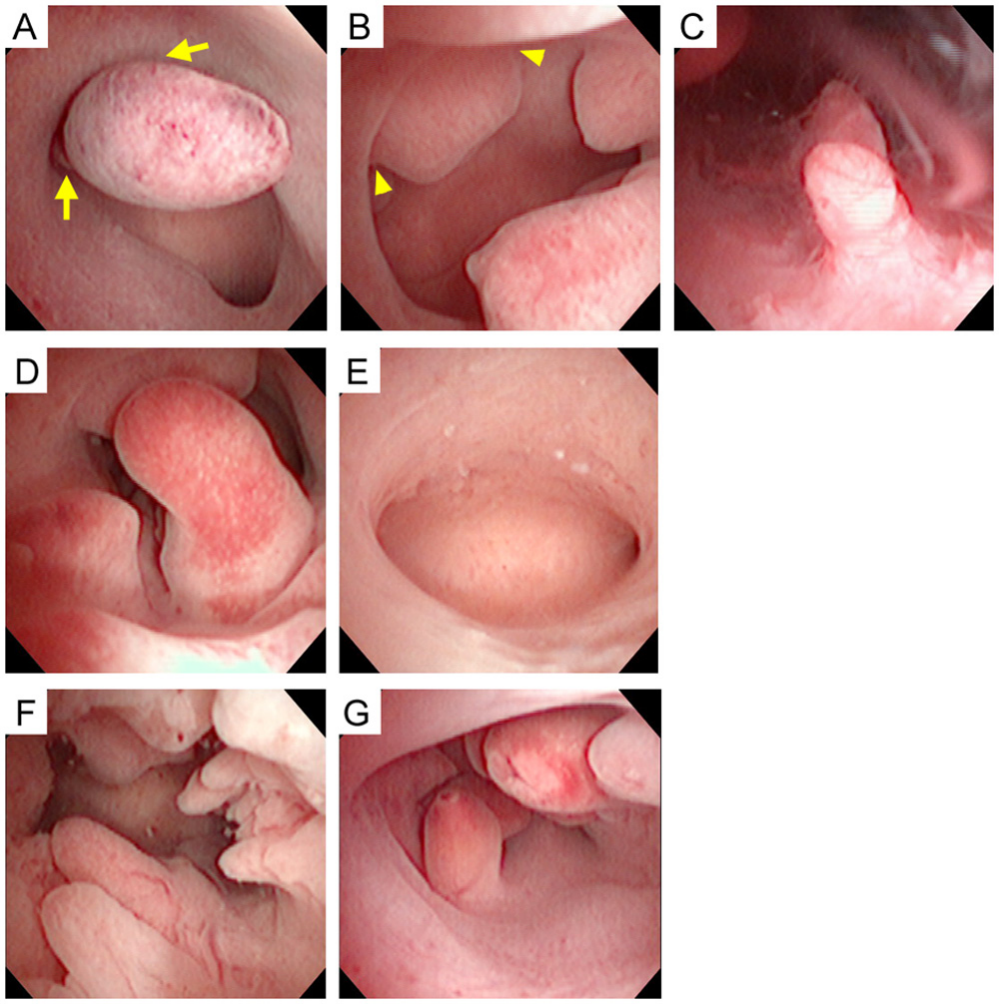
Office hysterosocpic examination of endometrial polyps. **(A)** Pedunculated endometrial polyps are defined with the angle of polyp surface to endometrium < 90 degrees (arrow). **(B)** Sessile endometrial polyps are shown with an angle ≥ 90 degrees (arrowhead). **(C)** Presence of intracavitary bleeding is noted. **(D, E)** A woman has three endometrial polyps, and the follow-up hysteroscopy does not find polyp recurrence 3 months after polypectomy. **(F, G)** Another woman has ten endometrial polyps, and postoperative hysteroscopy reveals polyp recurrence 8 months after polypectomy.

The number of endometrial polyps was counted, and their types, either pedunculated or sessile, were observed. The type of endometrial polyps was determined based on the angle that is made between the polyp and the adjacent uterine wall. Pedunculated type polyps were defined with the angle of polyp surface to endometrium < 90 degrees (Fig 1A), whereas sessile type polyps were those with an angle ≥ 90 degrees (Fig 1B). The presence of intracavitary bleeding could also be observed (Fig 1C). Office hysteroscopy was also done postoperatively for observation of the presence (Fig 1F, 1G and S2 Fig) or absence (Fig 1D, 1E and S1 Fig) of polyp recurrence.

### Hysteroscopic surgery

Hysteroscopic polypectomy was carried out in the follicular phase of the subsequent menstrual cycle. Operations were done exclusively by the same physician (JH Yang) with a 12-degree resectoscope bearing an outer diameter of 8 mm (Olympus Optical Co., Tokyo, Japan) [13,14,15]. The surgery was undertaken using a cutting-loop without the application of diathermy. All visible endometrial polyps were bluntly removed under direct hysteroscopic vision [16]. The reason why we did not use diathermy was to avoid deep injury to the endometrium or even myometrium as well as to prevent the formation of intrauterine adhesions, both of which might be unfavorable to future reproduction.

### Statistical analysis

Data were expressed as either median (25%-75%) or mean ± SD. Differences between the groups were compared using Mann-Whitney *U* test for continuous variables, and Fisher’s exact test for categorical variables. Univariate logistic regression analysis was done to examine the association between the risk of polyp recurrence and polyp number, as well as between the risk of polyp recurrence and follow-up duration. Multivariate logistic regression analysis was performed, in which recurrence of endometrial polyp was used as the dependent variable, while age, type of endometrial polyp, polyp number, and follow-up duration were used as independent variables.

Since 25%, 50%, and 75% values of the polyp number in all subjects were 1, 3, and 5 respectively, we then subgrouped them into n = 1, between 2 and 3, between 4 and 5, and ≥ 6. Cochran-Armitage trend test and multilinear regression analysis with dummy variables were carried out to examine the trend of polyp recurrence risk with the incremental levels of follow-up duration and polyp number. Kruskal-Wallis test was done to examine differences among three groups. A *P* value < 0.05 was considered statistically significant. All statistical analyses were performed using the PC version of the Statistical Analysis System (SAS version 9.1; SAS Institute Inc., Cary, NC) and the Statistical Program for Social Sciences (SPSS version 12; SPSS Inc., Chicago, IL).

## Results

Clinical characteristics of 168 women in this study are shown in Table 1. Infertility (80%) and AUB (48%) were two major indications for hysteroscopic polypectomy, in which 48 women (29%) had both infertility and AUB. Two women (1%) had neither infertility nor AUB, but asked for polypectomy for fear of malignant change of endometrial polyps.

**Table 1:** Demographic data for women with and without postoperative recurrence of endometrial polyps.

	Recurrence (n = 73)	No recurrence (n = 95)	*P* value
Age (yr)	36 (32–40)^a^	37 (34–42)	0.325
TCR indication			
Infertility	54 (74%)^b^	80 (84%)^c^	0.122
AUB	38 (52%)^b^	42 (44%)^c^	0.351
Others	1 (1%)	1 (1%)	1.000
Gravida	0 (0–2)	0 (0–1)	0.784
Parity	0 (0–1)	0 (0–1)	0.839
Polyp type			0.588
Pedunculated	15 (21%)	26 (27%)	
Sessile	41 (56%)	47 (50%)	
Both	17 (23%)	22 (23%)	
Polyp No.	4 (2–6)	2 (1–4)	0.010
Follow-up duration			0.035
<1 yr	25 (34%)	45 (47%)	
1–2 yr	19 (26%)	30 (32%)	
2–3 yr	12 (16%)	12 (13%)	
>3 yr	17 (23%)	8 (8%)	

TCR = transcervical resection, AUB = abnormal uterine bleeding

^a^ Median (range 25–75%)

^b^ 20 women had both infertility and AUB.

^c^ 28 women had both infertility and AUB.

Pedunculated and sessile type polyps accounted for 24% and 52% of the cases respectively, and another 23% women had both types of endometrial polyps. The number of endometrial polyps were between 1 and 15 with a mean value of 3.6, in which women with a polyp recurrence had a higher number than those without polyp recurrence (median = 4 vs. 2, *P* = 0.010). The follow-up duration ranged from 2 to 73 months with a mean value of 18.8 months, and women with polyp recurrence had longer follow-up duration than those without polyp recurrence (median = 18 vs. 12 months, *P* = 0.035).

Pedunculated type endometrial polyps had a stronger association with AUB (*P* = 0.001 by Fisher’s exact test, Table 2) than sessile type polyps. Ninety-five women (57%) were free from polyp recurrence and 73 women (43%) had polyp recurrence that was confirmed by postoperative office hysteroscopy (Fig 1). For those with polyp recurrence, the comparison between pre- and post-operative hysteroscopic pictures revealed that 39 women (53%) had polyp recurrence at the same location, 17 women (23%) had polyp recurrence at different locations, and another 17 women (23%) had polyp recurrence at the same location as well as at different locations. The number of polyps was the only factor that determined the location of polyp recurrence (*P* = 0.009 by Kruskal-Wallis test, Table 3).

**Table 2:** Relationship between pedunculated type endometrial polyps and the presentation of abnormal uterine bleeding (AUB).

		Pedunculated type polyp	
		+	-
AUB	+	57	31
	-	31	49

*P* = 0.001 (Fisher’s exact test)

Odds ratio 2.906 (95% confidence interval 1.55–5.44, *P* = 0.0009) by Wald test

**Table 3:** Factors affecting the recurrence locations of endometrial polyps.

	Same location (n = 39)	Different locations (n = 17)	Both (n = 17)	*P* value^a^
Age	36.2 ± 5.4	39.4 ± 6.2	34.5 ± 6.5	0.109
Gravida	0.9 ± 1.2	0.7 ± 1.0	0.5 ± 0.8	0.523
Parity	0.4 ± 0.7	0.5 ± 0.8	0.4 ± 0.7	0.851
Polyp No.	5.0 ± 3.5	2.3 ± 1.6	4.8 ± 3.0	0.009
Follow-up (m)	22.3 ± 14.5	23.7 ± 16.4	22.3 ± 18.1	0.875

^a^ Kruskal-Wallis test

To further investigate the effect of polyp number on the postoperative polyp recurrence, we classified the polyp number into four groups, that is, number = 1, 2–3, 4–5, and ≥ 6. As the polyp number increased, there was a significant trend toward a higher risk of polyp recurrence (*P* = 0.017 by Cochran-Armitage trend test). When we used polyp number = 1 as the benchmark, the risks of polyp recurrence were 1.01, 1.83, and 2.62 respectively in women having a polyp number 2–3, 4–5, and ≥ 6. After adjustment for age, polyp type, and follow-up duration, the risk of polyp recurrence in relation to the number of endometrial polyps was even higher (odds ratio 1.33, 2.85, and 3.48 respectively as compared with those who had only one endometrial polyp, *P* for trend test = 0.015) (Table 4).

**Table 4:** Risk of polyp recurrence in women with different numbers of endometrial polyps.

	Endometrial polyp No.				*P* for trend
	1	2–3	4–5	≥6	
Recurrence	18/51	16/45	19/38	20/34	
	(35%)	(36%)	(50%)	(59%)	
Unadjusted OR (95% CI)	1	1.01 (0.44–2.34)	1.83 (0.78–4.32)	2.62 (1.07–6.39)	0.017
Adjusted OR (95% CI)^a^	1	1.33 (0.47–3.78)	2.85 (0.88–9.21)	3.48 (1.01–12.0)	0.015

OR = odds ratio, CI = confidence interval

^a^ Multivariate logistic regression analysis was applied to assess the association between endometrial polyp number and the risk of polyp recurrence after adjustment for age, polyp type, and follow-up duration.

With regard to the effect of follow-up duration on the postoperative polyp recurrence, the follow-up duration was classified into four groups, that is, < 1 year, 1–1.9 years, 2–2.9 years, and ≥ 3 years. As the follow-up duration was longer, a significant trend toward a higher risk of polyp recurrence was found (*P* = 0.005 by Cochran-Armitage trend test). As we used follow-up duration < 1 year as the benchmark, the risks of polyp recurrence were 1.14, 1.8, and 3.25 respectively in women having follow-up duration 1–1.9 years, 2–2.9 years, and ≥ 3 years. After adjustment for age, polyp type, and polyp number, the risk of polyp recurrence in relation to the follow-up duration was even higher (odds ratio 1.27, 2.33, and 3.92 respectively as compared with those having a follow-up duration < 1 year, *P* for trend test = 0.004) (Table 5).

**Table 5:** Risk of polyp recurrence in women with different follow-up duration.

	Follow-up duration				*P* for trend
	<1 yr	1–1.9 yr	2–2.9 yr	≥3 yr	
Recurrence	25/70	19/49	12/24	17/25	
	(36%)	(39%)	(50%)	(68%)	
Unadjusted OR (95% CI)	1	1.14 (0.54–2.42)	1.80 (0.71–4.60)	3.25 (1.45–10.11)	0.005
Adjusted OR (95% CI)^a^	1	1.27 (0.58–2.79)	2.33 (0.86–6.26)	3.92 (1.41–10.9)	0.004

OR = odds ratio, CI = confidence interval

^a^ Multivariate logistic regression analysis was applied to assess the association between follow-up duration and the risk of polyp recurrence after adjustment for age, polyp type, and polyp number.

## Discussion

This is a study investigating the factors influencing the postoperative recurrence potential of benign endometrial polyps. After considering all the variables including age, polyp type, polyp number and follow-up duration, the multivariate regression model revealed that polyp number and follow-up duration, but not age and polyp type, were significantly associated with the risk of postoperative polyp recurrence. Our study focuses on benign endometrial polyps, and their recurrence potential increases as the follow-up duration is longer. This positive association between the recurrence rate and follow-up period is also found in endometriosis, in which the 2-year recurrence rate is 21.5% postoperatively, whereas the 5-year recurrence rate is as high as 40–50% [17].

The type of endometrial polyps, either pedunculated or sessile, does not play a role in the postoperative recurrence potential, whereas pedunculated polyps are strongly associated with the presentation of AUB. The uterine cavity is a potential but collapsed cavity in normal conditions. Compared with sessile polyps, the surface of pedunculated endometrial polyps is prone to touch the endometrium of the opposite uterine wall, and AUB occurs consequently. The intracavitary bleeding might therefore interfere with embryo implantation [2].

The postoperative polyp recurrence rate was 43% in this study, higher than those reported previously [3,8,9]. There are three possible reasons accounting for this relatively high recurrence rate. First, this is a retrospective study, and we only included women who underwent office hysteroscopy both pre- and postoperatively. Women who didn’t achieve a pregnancy and/or those who suffered from recurrence of AUB were more likely to ask for an additional hysteroscopic examination, and they bore a higher risk of polyp recurrence. By contrast, those who successfully achieved a pregnancy and/or those who were free from AUB after polypectomy were less likely to come back for an additional hysteroscopic examination. As a result, the recurrence rate would be high in this study because we only enrolled the high-risk population.

Secondly, we performed hysteroscopic polypectomy without the application of diathermy. All visible endometrial polyps were bluntly removed with the cutting-loop under direct hysteroscopic vision. The reason why we did not use diathermy was to avoid deep injury to the endometrium or even myometrium as well as to prevent the formation of intrauterine adhesions, both of which might be unfavorable to future reproduction. Accordingly, the stalk and base of endometrial polyps might not be completely removed, which enhanced the risk of polyp recurrence. Comparatively, hysteroscopic resection using monopolar energy is highly effective in the treatment of endometrial polyps with a recurrence rate of 0–4.5% [6,8]. Thirdly, the mean number of endometrial polyps is 3.6 in this study, more than that reported in previous studies [3,8,9], and therefore the postoperative polyp recurrence rate would be higher.

The location of recurrent polyps was also evaluated with hysteroscopy in this study, and results revealed that a higher number of endometrial polyps were associated with an increased postoperative recurrence potential of polyps at the same location (Table 3). Although hysteroscopy is considered the gold standard for diagnosis of endometrial polyps [18], it is difficult to determine retrospectively if recurrence develops in a location near or far from the previous polyp [3]. In addition, a high number of polyps make this judgment even harder during hysteroscopic examinations.

Hysteroscopic polypectomy is presently the gold standard for treatment of endometrial polyps. It is undertaken under direct vision to completely remove the polyps while preserving the adjacent endometrium [3]. For infertile women with otherwise unexplained infertility, hysteroscopic polypectomy improves fertility and increases pregnancy rates [2,6,7]. Our previous study found that 86% of women had their endometrium heal within 1 month after hysteroscopic polypectomy, and another 14% achieved wound healing between one and two months [16]. As a result, infertile women should be instructed to achieve a pregnancy 1–2 months after hysteroscopic polypectomy, and as soon as possible before polyp recurrence.

## Conclusions

The number of endometrial polyps and follow-up duration, but not the type of polyps, are major factors that determine the recurrence potential after hysteroscopic polypectomy. A higher number of endometrial polyps and longer follow-up duration are associated with a greater potential of polyp recurrence.

## Supporting Information

S1 Fig A 44-year-old woman has two endometrial polyps, and the follow-up hysteroscopy does not find polyp recurrence 10 months after hysteroscopic polypectomy. (TIF)

S2 Fig A 36-year-old woman has one endometrial polyp, and the follow-up hysteroscopy detects polyp recurrence at the same location 14 months after hysteroscopic polypectomy. (TIF)
