# Supplementary figures and images for: Factors Influencing the Recurrence Potential of Benign Endometrial Polyps after Hysteroscopic Polypectomy
Source: PLoS One. 2015 Dec 11;10(12):e0144857. doi: 10.1371/journal.pone.0144857 (PMC4676604; doi:10.1371/journal.pone.0144857)

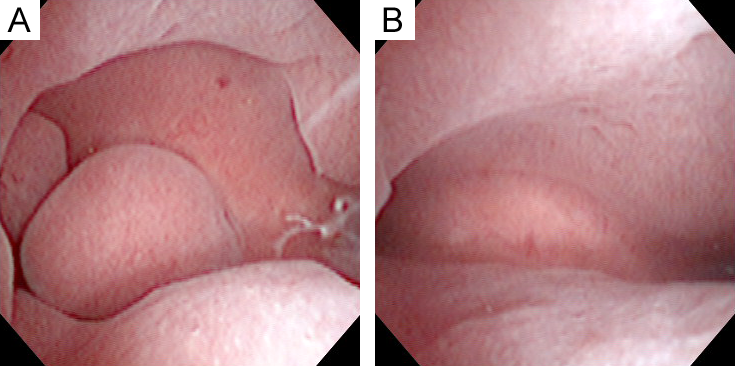

Supplement: S1 Fig — (TIF) [file pone.0144857.s001.tif]

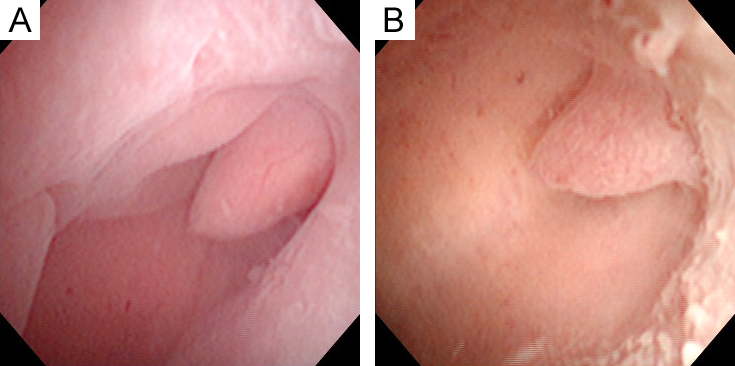

Supplement: S2 Fig — (TIF) [file pone.0144857.s002.tif]
